# Supplementary material for: Full-length isoform constructor (FLIC) – a tool for isoform discovery based on long reads
Source: Bioinformatics. 2025 Oct 1;41(12):btaf551. doi: 10.1093/bioinformatics/btaf551 (PMC12771368; doi:10.1093/bioinformatics/btaf551)
Supplement: btaf551_Supplementary_Data [file btaf551_Supplementary_Data.pdf]

Supplementary Files Content

**Figure S1**	The workflow of ONT library construction and ONT reads filtration
**Figure S2**	*Downsampling module* workflow in annotation-free mode
**Figure S3**	Isoform reconstruction quality
**Figure S4**	The intersection of isoforms obtained by FLIC and presented in *Arabidopsis* genome annotation for genes expressed in leaf sample
**Figure S5**	Illumina expression support of short isoforms initiating within introns
**Figure S6**	The comparison of gene length reconstructed by FLIC and presented in *Arabidopsis* genome annotation
**Table S1**	Mapping statistics of reads used in the study
**Table S2**	Statistics of splice sites detection in ONT data by splice sites identification module of FLIC
**Table S3**	Statistics of gene number downsampled by *downsampling module* of FLIC
**Table S4**	True positive (TP) rates for isoform reconstruction in the presence of distorted annotation and in annotation-free modes (pointes)
**Table S5**	True positive (TP) rates for isoform reconstruction in the presence of distorted annotation and in annotation-free modes (ranges)

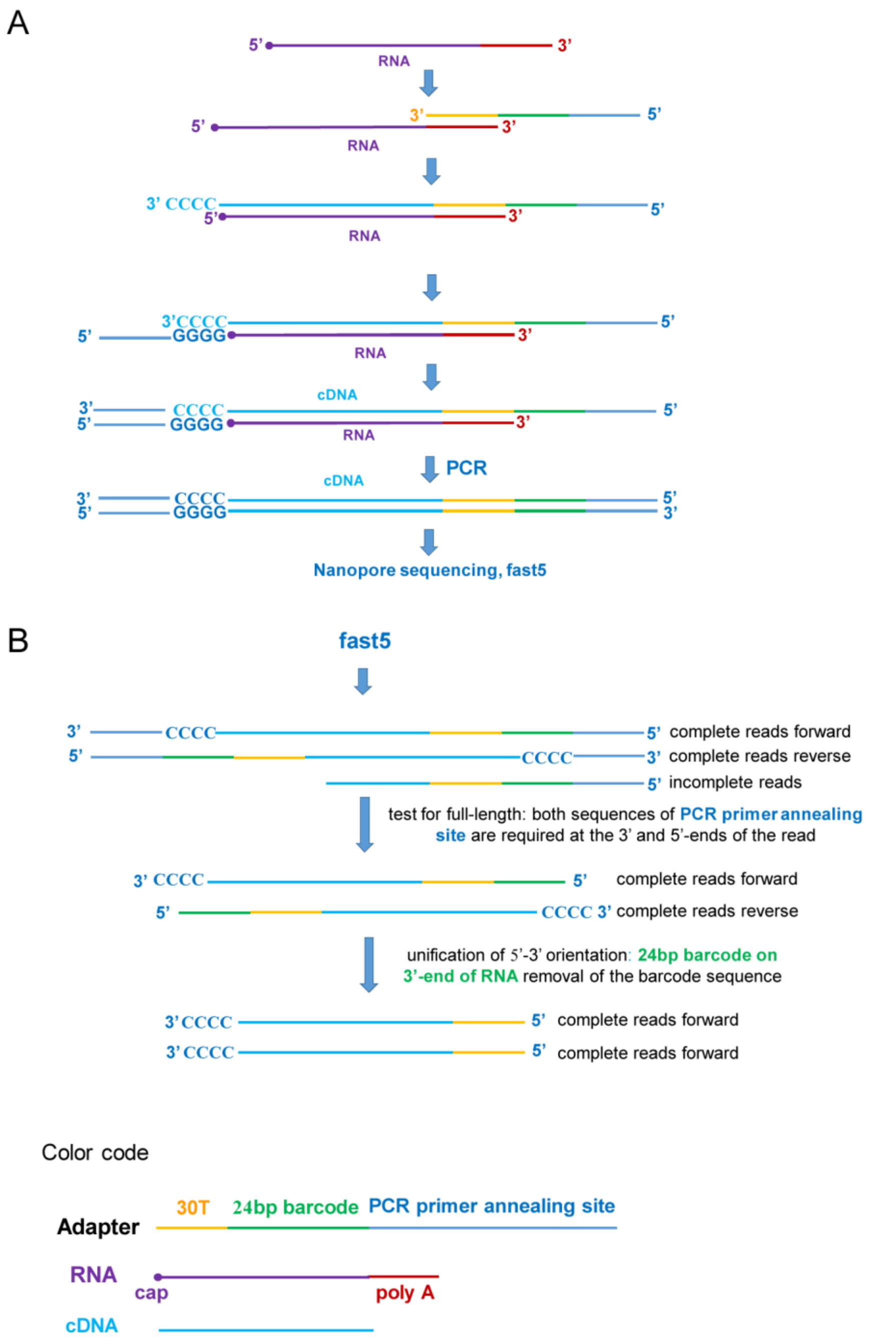

Supplementary Figure 1. The workflow of ONT library construction and ONT reads filtration. **(A)** The adaptation of SMART protocol. **(B)** Stages of ONT reads filtration.

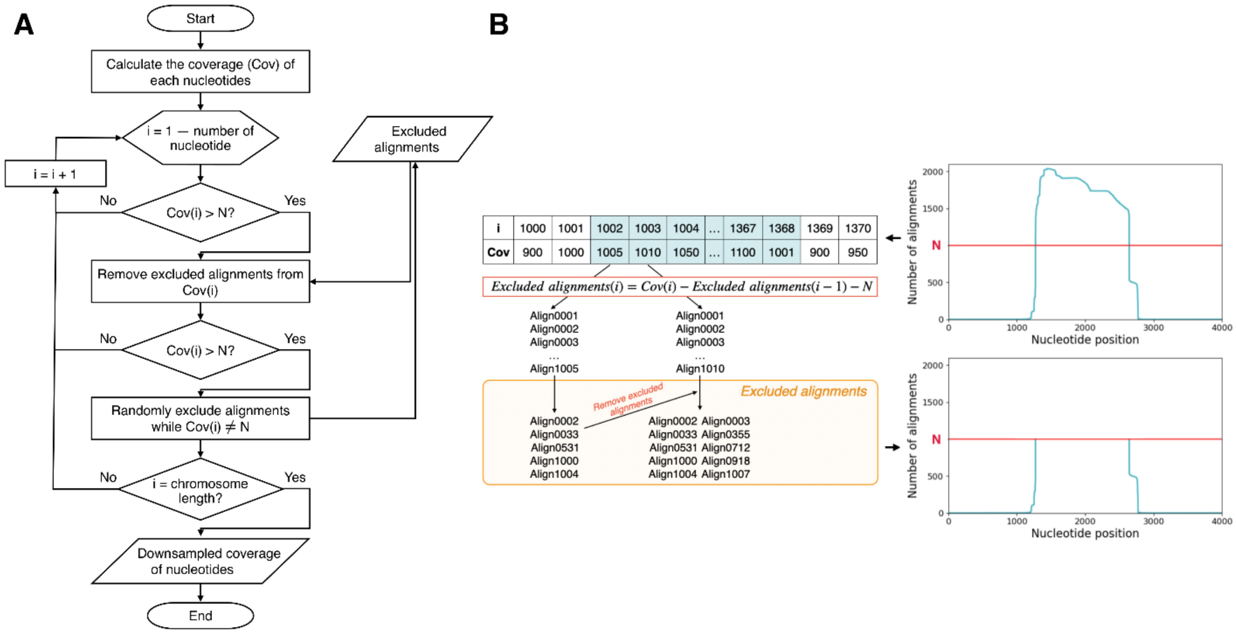

Supplementary Figure 2. *Downsampling module* workflow in annotation-free mode. **(A)** Flowchart of the module's operation. **(B)** Conceptual illustration showing the module's functionality during the downsampling process.

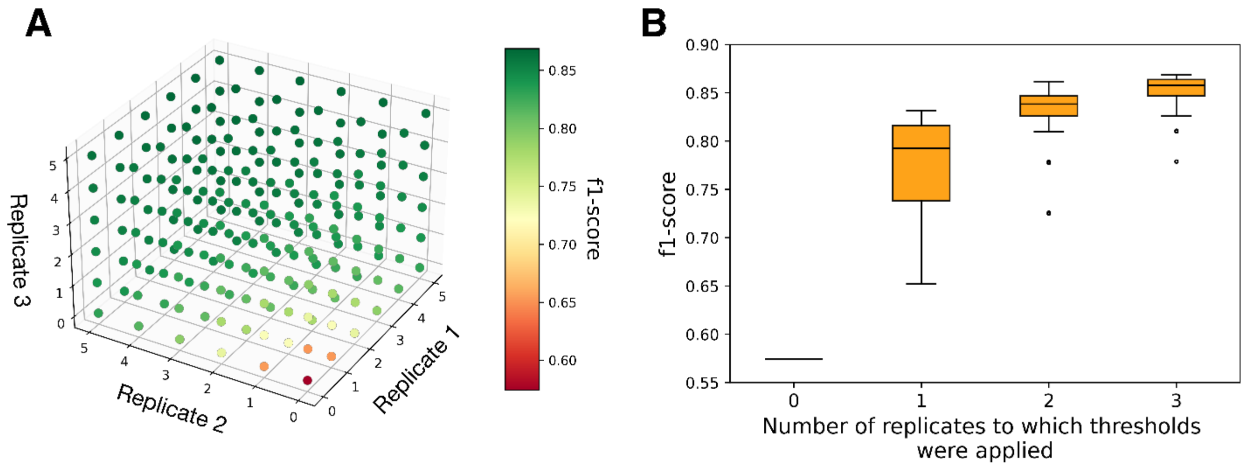

Supplementary Figure S3. Isoform reconstruction quality. (A) Dependence of isoform reconstruction quality on the number of replicates to which a read-count threshold is applied. Threshold values for the selected replicates ranged from 1 to 5 reads (B) Evaluation of the impact of read count threshold values on isoform reconstruction efficiency.

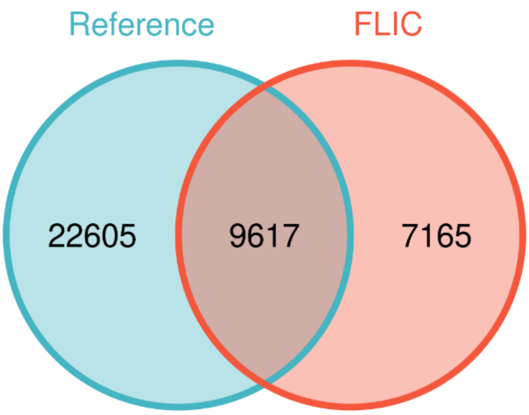

Supplementary Figure S4. The intersection of isoforms obtained by FLIC and presented in *Arabidopsis* genome annotation for genes expressed in leaf sample. TSSs and PAs were excluded from the analysis.

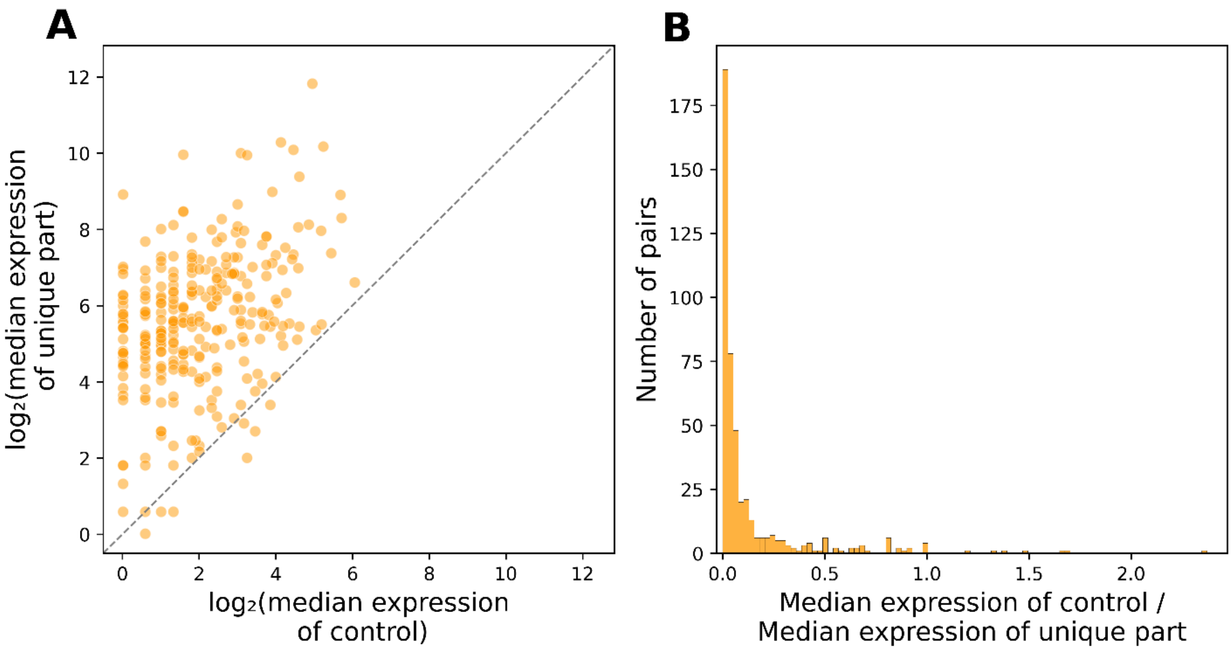

Supplementary Figure S5. Illumina expression support of short isoforms initiating within introns. (A) Log_2-transformed median coverage of regions of short isoforms versus the remaining parts of the same introns. (B) Distribution of coverage ratios of expression levels (control – part that is constitutively intronic/part that is included in short isoform). Ratios under 1 indicate higher expression in the regions of short isoforms.

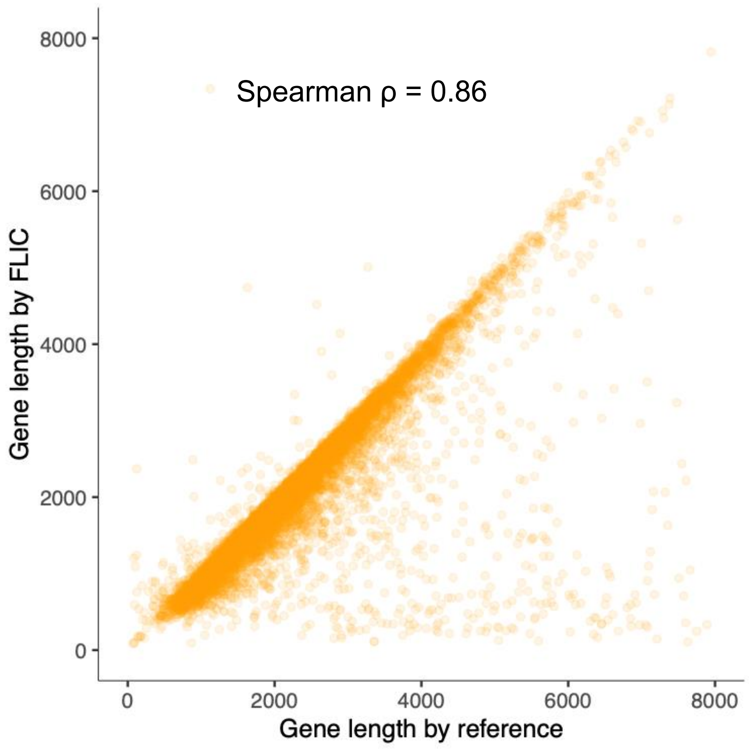

Supplementary Figure S6. The comparison of gene length reconstructed by FLIC and presented in *Arabidopsis* genome annotation.

Supplementary Table S1. Mapping statistics of reads used in the study

Three replicates of *Arabidopsis* leaves, ONT libraries

	Replicate 1	Replicate 2	Replicate 3
Raw read number	9 459 558	10 389 060	6 952 504
Number of read trimmed by qualitry and M1 primers presence (percentage of raw read number)	7 057 521 (75%)	7 405 646 (71%)	5 281 786 (76%)
Number of mapped reads (percentage of trimmed reads)	6 969 873 (99%)	7 284 457 (98%)	5 200 431 (98%)
Uniquely mapped reads (percentage of trimmed reads)	6 681 390 (95%)	6 742 474 (91%)	4 985 049 (94%)

Three replicates of *Arabidopsis* leaves, CAGE libraries

	Replicate 1	Replicate 2	Replicate 3
Raw read number	50 177 822	45 187 079	40 547 826
Number of read trimmed by qualitry (percentage of raw read number)	48 491 378 (97%)	42 463 180 (94%)	38 909 790 (96%)
Number of mapped reads, primary alignments only (percentage of trimmed reads)	47 215 840 (97%)	41 363 934 (97%)	37 910 884 (97%)

Supplementary Table S2. Statistics of splice sites detection in ONT data by splice sites identification module of FLIC

	Replicate 1	Replicate 2	Replicate 3
Number of reference splice sites identified in ONT reads (percentage of total number (254 309) of splice sites in Illumina data	112 623 (44.3%)	114 608 (45.1%)	97 477 (38.3%)
Total number of prolonged gaps in ONT reads	14 915 516	14 769 888	10 423 633
Number of ONT gaps perfectly matched with reference splice sites (percentage of total number)	13 803 535 (92.5%)	13 645 115 (92.4%)	9 730 327 (93.3%)
Number of ONT gaps intersected with reference splice sites (percentage of total number)	877 525 (5.9%)	876 276 (5.9%	596 516 (5.7%
Number of ONT gaps not matched with reference splice sites (percentage of total number)	234 456 (1.6%)	248 497 (1.7%)	96 790 (0.9%)

Supplementary Table S3. Statistics of gene number downsampled by downsampling module of FLIC

	Replicate 1	Replicate 2	Replicate 3
Total number of genes covered by at least five ONT reads	16 810	17 322	16 250
Number of genes for which downsampling was applied (percentage of total number)	719 (4.3%)	761 (4.4%)	577 (3.6%)
Number of genes without downsampling (percentage of total number)	16 091 (95.7%)	16 561 (95.6%)	15673 (93.4%)

Supplementary Table S4. True positive (TP) rates for isoform reconstruction in the presence of distorted annotation and in annotation-free modes. TSSs and PAs were treated as points (nucleotide position with maximal coverage inside corresponding range)

Type of gene annotation disturbance	Number of genes	FLIC, % TP	Isoquant, % TP	StringTie, % TP	TALON, % TP	FLIC anno-free, % TP	Isoquant anno-free, % TP	StringTie anno-free, % TP
Unchanged genes	7 177	77.2%	79.4%	46.7%	94.3%	76.9%	28.4%	26.9%
5' shortage	7 264	76.3%	3.1%	10.6%	0.0%	76.5%	29.1%	27.4%
3' shortage	7 232	76.8%	1.0%	20.3%	0.0%	77.2%	29.3%	27.9%
5' elongation	7 274	76.3%	0.0%	35.7%	0.0%	75.9%	28.4%	27.3%
3' elongation	7 217	77.4%	0.0%	27.1%	0.0%	76.9%	28.5%	27.3%
5' and 3' shortage	7 233	76.3%	3.4%	20.2%	0.0%	76.1%	28.8%	26.8%
5' and 3' elongation	7 162	77.9%	0.0%	25.7%	0.0%	77.6%	28.3%	27.7%

Supplementary Table S5. True positive (TP) rates for isoform reconstruction in the presence of distorted annotation and in annotation-free modes. TSSs and PAs were treated as ranges.

Type of gene annotation disturbance	Number of genes	FLIC, % TP	Isoquant, % TP	StringTie, % TP	TALON, % TP	FLIC anno-free, % TP	Isoquant anno-free, % TP	StringTie anno-free, % TP
Unchanged genes	7 177	97.2%	79.4%	55.1%	94.2%	97.2%	43.7%	37.2%
5' shortage	7 264	96.4%	6.3%	13.6%	1.8%	96.8%	44.3%	37.4%
3' shortage	7 232	96.8%	1.3%	27.2%	0.1%	97.3%	43.6%	38.4%
5' elongation	7 274	97.2%	0.7%	47.7%	1.7%	97.2%	43.2%	38.0%
3' elongation	7 217	97.1%	0.0%	41.5%	0.1%	97.1%	43.0%	37.6%
5' and 3' shortage	7 233	97.1%	4.9%	28.2%	0.1%	97.2%	44.1%	37.7%
5' and 3' elongation	7 162	97.4%	0.0%	39.7%	0.1%	97.4%	43.1%	38.2%
